# Diurnal Rhythmicity in the Rhizosphere Microbiome—Mechanistic Insights and Significance for Rhizosphere Function

**DOI:** 10.1111/pce.15283

**Published:** 2024-11-18

**Authors:** Gary D. Bending, Amy Newman, Emma Picot, Ryan M. Mushinski, Davey L. Jones, Isabelle A. Carré

**Affiliations:** ^1^ School of Life Sciences University of Warwick Coventry UK; ^2^ School of Environmental and Natural Sciences Bangor University Bangor UK; ^3^ Food Futures Institute Murdoch University Perth WA Australia

## Abstract

The rhizosphere is a key interface between plants, microbes and the soil which influences plant health and nutrition and modulates terrestrial biogeochemical cycling. Recent research has shown that the rhizosphere environment is far more dynamic than previously recognised, with evidence emerging for diurnal rhythmicity in rhizosphere chemistry and microbial community composition. This rhythmicity is in part linked to the host plant's circadian rhythm, although some heterotrophic rhizosphere bacteria and fungi may also possess intrinsic rhythmicity. We review the evidence for diurnal rhythmicity in rhizosphere microbial communities and its link to the plant circadian clock. Factors which may drive microbial rhythmicity are discussed, including diurnal change in root exudate flux and composition, rhizosphere physico‐chemical properties and plant immunity. Microbial processes which could contribute to community rhythmicity are considered, including self‐sustained microbial rhythms, bacterial movement into and out of the rhizosphere, and microbe‐microbe interactions. We also consider evidence that changes in microbial composition mediated by the plant circadian clock may affect microbial function and its significance for plant health and broader soil biogeochemical cycling processes. We identify key knowledge gaps and approaches which could help to resolve the spatial and temporal variation and functional significance of rhizosphere microbial rhythmicity. This includes unravelling the factors which determine the oscillation of microbial activity, growth and death, and cross‐talk with the host over diurnal time frames. We conclude that diurnal rhythmicity is an inherent characteristic of the rhizosphere and that temporal factors should be considered and reported in rhizosphere studies.

## Summary statement

We review evidence that the rhizosphere is a diurnally rhythmic interface between plants, microbes, and soil which is under the influence of the plant circadian clock. We highlight the importance of considering temporal factors when interpreting rhizosphere data.

## 1 The Significance of the Rhizosphere

**Figure 1:**
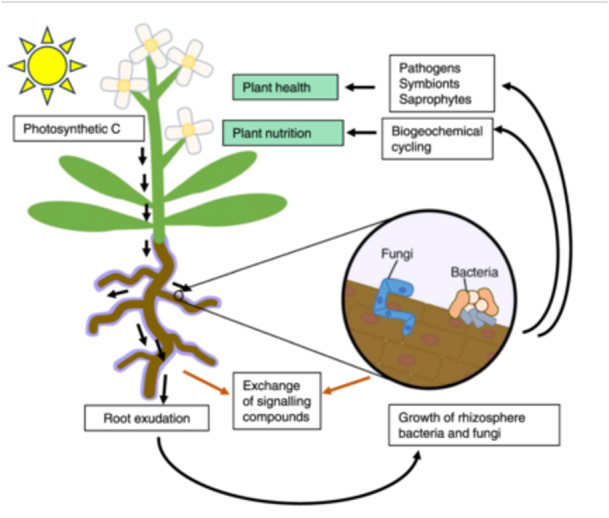
The rhizosphere represents the roots and soil adjacent to roots. Root exudation drives growth of microbes in the rhizosphere and these communities can interact with the plant and soil to direct biogeochemical cycling processes, and plant health and nutrition. The plant and microbial communities exchange a range of signalling compounds which influence each other's growth and physiology.

The rhizosphere encompasses the roots together with the adjacent zone of soil (Figure 1). It has long been recognised that rhizosphere soil represents a distinct physical and chemical environment relative to the bulk soil. This is the result of soil compaction as roots grow through it, the uptake of nutrients and water by plant roots, and the release of a wide range of carbon‐rich materials into the soil by plant roots, a process termed ‘rhizodeposition’ (Gregory 2006). These factors, particularly rhizodeposition, drive the recruitment of specific biota from the soil into both the rhizosphere soil and the root itself. The rhizosphere microbiome has been referred to as the plant's ‘second genome’ (Berendsen, Pieterse, and Bakker 2012), as its components act to broaden the functional repertoire of plants and are a key determinant of plant health (Bakker et al. 2013). The rhizosphere is therefore a dynamic interface which simultaneously facilitates plant‐microbe interactions while also acting as a major conduit for biogeochemical cycling between the atmosphere, plant and soil in terrestrial ecosystems.

Rhizodeposition occurs via several defined pathways. Photosynthetic assimilates in the form of fatty acids and low molecular weight sugars such as sucrose can be transferred by the plant directly to root‐inhabiting rhizosphere symbionts (e.g. rhizobia and mycorrhizal fungi) in exchange for nutrients (Udvardi and Kahn 1993; Simard, Jones, and Durall 2003; Jiang et al. 2017). Plant carbon (C) is also deposited into rhizosphere soil as cells are sloughed off the root tip as a result of growth and following root senescence. Importantly, 5%–10% of the C fixed by photosynthesis is exuded into the soil as diverse compounds, including mucilage, organic acids, amino acids, sugars and secondary metabolites. Roots may also release antimicrobial compounds to suppress microbial activity in the surrounding soil (Kocyigit et al. 2023). These materials can either be exuded actively through transporters, or passively by diffusion. Exudate quantity and composition are determined by a host of biotic and abiotic factors including plant species, age, soil properties and climate. The microbial community inhabiting the rhizosphere may also promote exudation by continually removing exudates to intensify the diffusion gradient or by altering the permeability of the root membrane, thus increasing the flow and diversity of C compounds entering the soil (Jones, Nguyen, and Finlay 2009).

Because of the availability of root‐derived C, microbial biomass in rhizosphere soil can be orders of magnitude higher than in the surrounding bulk soil (Fierer 2017). The quality and quantity of rhizodeposits is a key determinant of the microbial community which assembles in the rhizosphere. This is further influenced by a wide range of factors including climate, soil properties and agricultural management practices, which together shape the soil microbial community from which rhizosphere biota are recruited (Hilton et al. 2021; Picot et al. 2021). As a result of the complex interactions which determine rhizodeposition, soil community composition and recruitment of biota into both the root and rhizosphere soil, it is increasingly recognised that rhizosphere microbiome composition is dynamic across time, and can change across seasons and between years (Barnes et al. 2016; Barnes et al. 2018).

The composition of the rhizosphere microbiome can have profound impacts on a wide range of plant characteristics including nutrition, morphology and productivity. In particular, communities can act as a continuum from beneficial to harmful (Bennett et al. 2012; Hilton et al. 2021). This reflects the wide range of functional characteristics that rhizosphere microbiota can possess such as the potential to interact directly with the plant as mutualistic symbionts and pathogens, the capacity to prime plant defence pathways, the complement of biogeochemical cycling functions which determine the availability of nutrients in the root zone and the potential to affect other microbes and indirectly influence their interactions with the plant and soil. Since temporal variation in community composition is an inherent feature of the rhizosphere, it is likely that functional interactions within the microbiome which can influence plant health are also dynamic over time.

## 2 Diurnal Rhythmicity in Rhizosphere Microbiome Composition

Recent studies have demonstrated that the composition of the rhizosphere microbiome shows variation across diurnal time frames, indicating that the rhizosphere is a far more dynamic microbial environment than previously understood. Most research in this area has used amplicon sequencing (also known as ‘metabarcoding’). In this process, DNA or RNA is extracted from a root or rhizosphere soil microbial community, a taxonomic marker gene specific to the microbial group of interest is amplified by PCR, and the resulting amplicons are sequenced. Sequences are then either grouped into operational taxonomic units (OTU), typically at 97% similarity to approximate species or analysed as amplicon sequence variants (ASVs), which represent sequences with single nucleotide differences and provide finer taxonomic resolution, sometimes down to the strain level. ASV analysis highlights more subtle changes in communities and allows more direct comparisons between studies. OTU or ASV are used to profile the diversity and composition of communities but measure relative rather than absolute abundance of microbes. Changes in microbial community composition detected using these methods may reflect shifts in the relative growth or death of microbial OTU or ASV (i.e., change in cell number) within the community. When RNA is profiled rather than DNA, changes in the metabolic activity of microbiota can be detected, such as cells transitioning from dormant to active states.

Diel changes in rhizosphere microbial communities were first demonstrated in *Arabidopsis thaliana* and *Brachypodium distachyon* (Staley et al. 2017), in which there were significant differences in bacterial community composition between rhizosphere samples collected during the day and the night. Approximately 10% of the bacterial OTUs, showed diurnal fluctuations in their relative abundances (within the total bacterial community) in the Arabidopsis rhizosphere, while 3.5% of bacterial OTUs exhibited diurnal shifts in the *Brachypodium distachyon* rhizosphere. Rhythmicity in the composition of rhizosphere soil bacterial populations was subsequently confirmed in Arabidopsis (Hubbard et al. 2018; Lu et al. 2021; Newman et al. 2022), rice (Zhao et al. 2021) and the mustard *Boechera stricta* (Hubbard et al. 2023). The proportion of bacterial taxa which demonstrated rhythmicity in these studies ranged between 3‐5% (Hubbard et al. 2018; Newman et al. 2022; Hubbard et al. 2023) and 12%–14% (Lu et al. 2021; Zhao et al. 2021) at the lower and upper ends respectively in wild‐type plants. These studies have consistently shown that a wide range of bacterial taxa from multiple phyla can show rhythmicity. Furthermore, Newman et al. (2022) demonstrated that fungal communities also show marked diurnal changes in composition in the rhizosphere of Arabidopsis and that while most rhythmic fungi belonged to the Ascomycota, they belonged to diverse classes (Newman et al. 2022). Although only 6.2% of fungal taxa were rhythmic, the relative abundance of these taxa was 42%, suggesting that rhythmic changes may be particularly intense within the fungal community.

Many microbial cells which inhabit soil are dormant, and furthermore, soil contains considerable amounts of relic extracellular DNA which is stabilised for periods of time within the soil matrix following cell death (Carini et al. 2016). Communities profiled using DNA therefore reflect living, dormant and historic populations. Early studies of rhizosphere microbial rhythmicity used DNA extracts to profile microbial communities (Staley et al. 2017; Hubbard et al. 2018), whereas later studies used RNA to identify transcriptionally active populations (Zhao et al. 2021; Newman et al. 2022). Newman et al. (2022) found that analyses based on DNA and RNA identified similar proportions of rhythmic OTUs (3–5% in the rhizosphere of wild‐type plants), but the specific OTUs identified as rhythmic were distinct. This could suggest differences in the relative contributions of growth and metabolic activity to diurnal rhythmicity across the microbial community.

Most studies which have investigated diurnal changes in rhizosphere microbial composition have quantified OTU abundance relative to the total community. Therefore, the extent to which differences are the result of changes in absolute abundance or metabolic activity is unclear. However, Hubbard et al. (2023) showed that total DNA concentrations (combined for all microorganisms) in rhizosphere soil were three times greater in the daytime than those detected at night. This indicates that rhythmicity is associated with changes in microbial abundance, which could be explained by microbial growth, death and movement. Furthermore Zhao et al. (2021) quantified rhizosphere soil 16S rRNA abundance in cDNA over diurnal cycles and detected increased abundance in samples collected in the day relative to the night, indicating diurnal changes in transcriptional activity.

There is limited understanding of the functional significance of rhizosphere microbial rhythmicity. Staley et al. (2017) investigated the predicted metagenome associated with day and night‐time communities in the Arabidopsis rhizosphere, and this indicated significant differences in carbohydrate, lipid and amino acid metabolism. Similarly, Baraniya et al. (2018) performed low‐depth transcriptional profiling of *Hordeum vulgare* rhizosphere soil and found that transcriptional activity of amino acid and carbohydrate metabolism differed significantly between pre‐ and post‐dawn sampling points. This was associated with changes in abundance of bacterial groups including Burkholderiales, Caulobacterales, Rhizobiales, Sphingomonadales, and Xanthomonadales and the protist order Plasmodiophorida.

## 3 Plant and Microbial Circadian Clocks

Circadian clocks are circa‐24 h timing mechanisms which allow organisms to anticipate predictable changes in environmental conditions which result from the day–night cycle. This adaptation to the rhythmic nature of the environment has been found in a broad range of organisms ranging from animals and humans to plants and fungi as well as cyanobacteria (Young and Kay 2001). Much research has focused on the role of the plant circadian clock in directing the diurnal rhythmicity of rhizosphere biota. Mutations which alter the function of the plant host circadian clock have been shown to influence the composition of communities that assemble in the rhizosphere over the lifetime of a plant (Staley et al. 2017; Hubbard et al. 2018; Lu et al. 2021; Newman et al. 2022). Furthermore, while similar numbers of bacterial and fungal taxa showed diurnal rhythmicity in the rhizosphere of clock mutants, the rhythmicity of these taxa was shown to be specific to an individual mutant, with these taxa being arrhythmic in another mutant and in the wild‐type (Newman et al. 2022).

The persistence of daily rhythms following transfer to constant environmental conditions is generally taken as evidence of control by an endogenous circadian clock. Several studies have investigated the link between microbial rhythmicity and the plant circadian clock by transferring Arabidopsis plants grown under diel light–dark cycles to constant light (Newman et al. 2022) or dark (Zhao et al. 2021) conditions. While 12.2% of bacterial OTU showed rhythmicity under light–dark cycles, a comparable proportion (7.4%) maintained rhythmicity under constant dark conditions (Zhao et al. 2021). Similarly, diurnal rhythmicity persisted under constant light conditions for 1.6% and 12% of bacterial and fungal taxa (Newman et al. 2022).

Some diurnal rhythmicity in the abundance of rhizosphere biota may reflect the function of fungal or bacterial circadian clocks. The mechanism of the fungal circadian clock has been studied extensively in *Neurospora crassa*, a Sordariomycete, but evidence of circadian rhythmicity has also been found in a wide range of other fungi (Hevia et al. 2015). While circadian rhythmicity was long thought to be restricted to cyanobacteria (Eelderink‐Chen et al. 2021), several heterotrophic bacterial taxa have also been found to exhibit daily changes in abundance in bulk soil in constant darkness (Zhao et al. 2021), suggesting that some heterotrophic bacteria may also exhibit self‐autonomous rhythmicity in the soil and rhizosphere environment. This was further supported by evidence which showed that *Bacillus subtilis*, a common soil and rhizosphere inhabitant, exhibited ca‐24‐h changes in the expression of a luciferase reporter gene (Eelderink‐Chen et al. 2021). These oscillations exhibited the canonical properties of circadian rhythms, including entrainment to diurnal light‐dark cycles, persistence in constant darkness and temperature compensation (Sartor et al. 2023). However, nothing is currently known about the physiological significance of these microbial rhythms in the context of the rhizosphere.

## 4 Plant Processes Driving Microbial Rhythmicity

**Figure 2:**
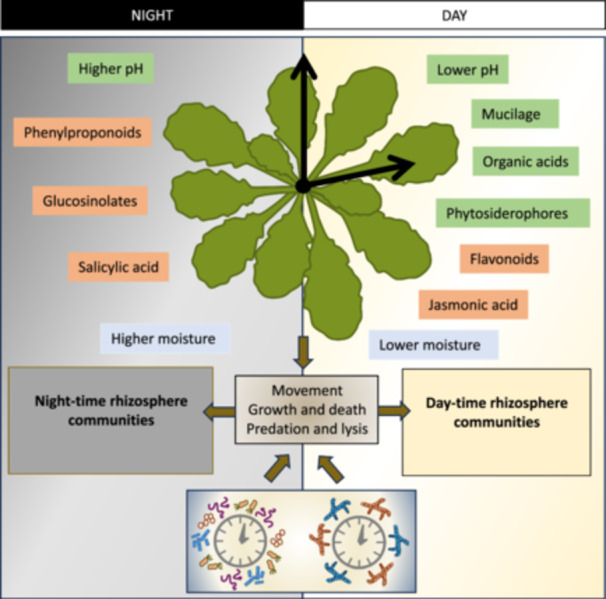
Potential drivers of microbial rhythmicity in the plant rhizosphere. Changes linked to plant root exudation are highlighted in green and those linked to increased plant transpiration during the day are highlighted in blue. Rhythmically produced molecules with roles in plant defence or plant‐microbe interactions are shown in orange. Some rhizosphere bacteria and fungi may also possess a circadian clock which could directly control rhythmic growth and/or activity. [Color figure can be viewed at wileyonlinelibrary.com]

The rhizosphere environment is modulated by the plant through many direct and indirect interactions which have the potential to influence the growth, activity and movement of microbial communities, thereby driving diurnal rhythmicity in the composition of microbial communities (Figure 2).

### 4.1 Rhythmic Changes in Root Exudation

The plant circadian clock drives many processes which may contribute to the creation of a rhythmic environment in the rhizosphere. However, plants may direct the diurnal rhythmicity of rhizosphere biota in a clock‐independent manner through direct effects of light‐dark or temperature cycles on root exudation. Photosynthetic C fixation is a rhythmic process, and the plant circadian clock is also involved in timing the transport, storage and metabolism of fixed C (de Barros Dantas et al. 2023). Diurnal changes in root exudation have been reported in several studies, although a direct linkage between exudation and the plant circadian clock has yet to be established. Studies indicate greater exudate flux during light relative to dark periods (Kuzyakov, Raskatov, and Kaupenjohann 2003; Melnitchouck et al. 2005). Similarly, elevated exudation of organic acids has been demonstrated during the day relative to the night (Watt and Evans 1999; Dessureault‐Rompré et al. 2007). Many studies have reported diel rhythms in the exudation of iron and zinc‐chelating phytosiderophores, which peak 2–6 h after the start of the light period (Zhang, Römheld, and Marschner 1991; Oburger et al. 2014). Similarly, exudation of the flavonoid catechin showed a diurnal rhythm, peaking 6 h after the start of the light phase (Tharayil and Triebwasser 2010). A small proportion of metabolites exuded from *A. thaliana* were found to follow diurnal cycles, peaking during the light period (Badri et al. 2010). The identity of these metabolites was not determined, but genes involved in the synthesis of phenylpropanoid and glucosinolate secondary metabolites were shown to display diel expression patterns, which increased during dark relative to light periods. In maize, mucilage exudation was diurnal and peaked at night, correlating with root elongation rate (Iijima, Sako, and Rao 2003). Both root and root hair growth are under the control of the circadian clock with growth being greater during the day (Narukawa, Watanabe, and Inoue 2010; Ikeda et al. 2023). As root cell expansion involves changes in membrane permeability, this may also result in diurnal, but highly spatially localised, shifts in root exudation. These circadian responses in specific root regions may be missed when soil from the whole root system is collected and analysed.

More recently, using untargeted metabolomics, Staley et al. (2017) found that the abundance of many compounds within the *A. thaliana* rhizosphere differed in abundance between light and dark periods, providing further evidence that plants exhibit diel differences in rhizodeposition. Here, a higher number of compounds were observed to be more abundant in samples taken during light relative to dark phases (Staley et al. 2017). Lipid‐like compounds dominated samples taken during dark phases, while compounds with higher oxygen:carbon ratios, which are likely to be flavonoids or organic acids, were more abundant in samples from the light period (Staley et al. 2017).

Within minutes of their release into the soil, low molecular weight exudates, such as organic acids, amino acids and sugars, are rapidly consumed by the soil microbial community (Gunina et al. 2017). These materials are further partitioned into new cell biomass and storage compounds, and may be respired within hours (Jones, Hodge, and Kuzyakov 2004). Daily variation in exudation rate is therefore likely to be a key determinant of C availability to microbiota in the rhizosphere. This may drive growth or activation of microbiota which utilise exudates during the day, when exudation rate peaks.

Plants may also express ultradian rhythms (cycles of ca. 1–4 h) which can be superimposed on circadian rhythms. These are of significance in root growth which is also likely to impact rates of rhizodeposition (Iijima and Matsushita 2011). The ultradian clock has also been implicated in ion flux oscillations in developing root hairs and nutrient uptake in roots (Simard, Jones, and Durall 2003).

### 4.2 Rhythmic Changes in Plant Immunity

Plant defence mechanisms are likely to be important in regulating the entry of rhizosphere microbiota into plant roots and their subsequent growth within root tissues. Rhythmic changes in plant immunity against pathogens have been demonstrated and this could also influence growth periods of endophytes including mycorrhizal symbionts (García‐Garrido and Ocampo 2002). Hernandez and Allen (2013) showed that the greatest rates of growth and subsequent dieback of extraradical hyphae in the rhizosphere occurred between 12:00 and 18:00 h in concert with the plant circadian clock. Similarly, arbuscular fungi have also been implicated in suppressing plant genes regulated by the circadian clock in response to drought stress, suggesting a close interplay between the plants and their symbionts in response to environmental stimuli (Ding et al. 2021).

*A. thaliana* shows rhythmic changes in susceptibility to multiple pathogens, including *Pseudomonas syringae*, *Botrytis cinerea*, and *Hyaloperonospora arabidopsidis* (Bhardwaj et al. 2011; Zhang et al. 2013; Hevia et al. 2015; Ingle et al. 2015). The accumulation of jasmonate and salicylate defence signalling hormones is regulated by the circadian clock, with jasmonate concentrations peaking in the subjective day and salicylates in the subjective night (Goodspeed et al. 2012). Jasmonic acid and salicylic acid are known to mediate defence responses to necrotrophic and biotrophic pathogens, respectively (Koornneef and Pieterse 2008; Bari and Jones 2009). Their accumulation at opposite phases of the diurnal cycle may therefore drive the alternance of distinct microbial populations in the root and rhizosphere. While jasmonic acid was found to be involved in bringing about rhythmic immunity against *B. cinerea* infection, peaking at dawn, Hevia et al. (2015) and, Ingle et al. (2015) showed that a circadian clock within *B. cinerea* itself was responsible for maximum disease severity in response to infection at night. This supports the argument that circadian rhythmicity in rhizosphere microbes may also contribute to the diurnal changes in rhizosphere microbial community composition. Rhythmic production of flavonoids may also be important since genes with roles in flavonoid biosynthesis show peak expression in the morning in both Arabidopsis and Medicago (Harmer et al. 2000; Achom et al. 2021). Many plant‐derived flavonoids are known to have antimicrobial activities, while others have signalling functions in the establishment of symbiotic interactions with nitrogen‐fixing rhizobia or arbuscular mycorrhizal fungi (Bag et al. 2022).

### 4.3 Rhythmic Changes in Mineral‐Nutrient Availability

There is some evidence that mineral nutrient uptake by plants may exhibit diel patterns (York et al. 2016), with the uptake of nitrate and potassium peaking during the light period and declining during the dark phase (Cardenas‐Navarro, Adamowicz, and Robin 1999; Kirkby et al. 2009). Many plant genes with roles in nutrient uptake exhibit rhythmic expression (Haydon, Bell, and Webb 2011). Transcripts for chloroplast sulphate and phosphate importers (Versaw and Harrison 2002; Cao et al. 2013) peak around dawn (Dodd et al. 2007; Covington et al. 2008; Wang et al. 2011) consistent with increased demands for photosynthesis during the day. Transcripts encoding proteins for nitrate (Ho et al. 2009) and ammonium uptake (Gazzarrini et al. 1999) have peak expression around dawn whereas transcripts for phloem loading of nitrate peak after dusk to drive source‐to‐sink movement of N in the night (Fan et al. 2009). Diurnal patterns in the uptake of nutrients by plant roots combined with diurnal changes in rhizosphere C availability as a result of exudation could drive steep temporal gradients in stoichiometry, which could exert selective pressures on rhizosphere microbes, and drive rhythmic changes in growth rate and composition.

### 4.4 Rhythmic Changes in the Physico‐Chemical Environment

Plant functions influence the physico‐chemical environment in rhizosphere soil, and changes over diurnal cycles may contribute to daily rhythmicity in microbial growth and movement. Plants show diurnal patterns of hydraulic conductance and root water content, controlled by the plant circadian clock (Takase et al. 2010; Caldeira et al. 2014), and this may drive rhythmic changes in rhizosphere moisture content (Montaldo and Oren 2022). Plant responses to salinity stress, particularly the upregulation of antioxidative mechanisms, have also been shown to have a circadian rhythm, potentially related to root growth and rhizodeposition (Venkat, Bae, and Muneer 2023). Diel oscillations in rhizosphere pH have been observed in several studies, with acidification during the light phase, and this may be linked to diurnal patterns of organic acid exudation and H^+^ release associated with nitrate uptake (Blossfeld et al. 2013; Rudolph et al. 2013; Zhao et al. 2021). Diurnal changes in oxygen levels were reported in the rhizosphere of rice plants, with anaerobic conditions observed during the night and oxygenated conditions during the day (Zhao et al. 2021).

## 5 Role of Microbial Processes

### 5.1 Microbial Movement

Bacteria possess a range of motility mechanisms using flagella, pili, gyration and sliding, which are used for chemotaxis towards favourable conditions in heterogeneous environments (Wadhwa and Berg 2022). Bacterial speeds of over 100 µm s^−1^ in aqueous media have been recorded, although the distances that could be achieved in more tortuous soil pore environments are less clear. However, many studies have shown chemotactic movement of bacteria towards the root in response to gradients of exudates such as organic acids (Zhang et al. 2014). Furthermore, salicylic acid (Section 4.2) has been found to inhibit the motility and growth of *Pseudomonas aeruginosa* by decreasing expression of *fliC*, which is involved in the production of flagellin, an important component of the flagellum required for motility (Dong et al. 2012). Changes in the relative abundance of bacteria within the rhizosphere over diurnal timescales may therefore reflect movement into and out of the rhizosphere zone, potentially driven by rhythmic salicylic acid production by the plant host. Studies have also indicated that root‐knot nematodes (*Meloidogyne* spp.) may also preferentially penetrate the root at night in response to plant circadian controls on growth and metabolism (Mishra and DiGennaro 2020), and evidence points to circadian regulation of sensory functions in nematodes (Olmedo et al. 2012). However, rhythmic movement would not account for changes in fungal relative abundance.

### 5.2 Microbial Interactions

The abundance and composition of microbial communities in the rhizosphere are likely to be strongly regulated by interactions with other biota, particularly microfauna and bacteriophages. The rhizosphere hosts a range of microfauna, including protists, nematodes, collembola and mites (Moore et al. 2003; Gao, Karlsson et al. 2018; Pratama et al. 2020; Hilton et al. 2021; Picot et al. 2021). These organisms have varied nutritional modes but include species which consume bacteria, fungi and other fauna. Protists and nematodes can exhibit distinct bacterial prey choices and exert top‐down regulation of communities with the potential to modulate rates of biogeochemical cycling processes (Gantner et al. 2006; Jiang et al. 2017; Gao, Karlsson et al. 2018). Understanding of the diversity and significance of bacteriophages in the rhizosphere is very limited, but recent evidence suggests that rhizosphere soil hosts distinct communities of DNA and RNA bacteriophages and patterns of phage activity relative to bulk soil (Muscatt et al. 2022), suggesting they are subject to strong selection pressures within the rhizosphere compartment. Studies of microbial rhythmicity in the rhizosphere have focussed on bacterial and fungal communities, and the extent to which they operate in other microbial groups, and the potential for microbe‐microbe interactions to contribute to patterns of microbial rhythmicity, are unclear.

## 6 Functional Significance of Microbial Rhythmicity in the Rhizosphere

Several studies have compared bacterial (Staley et al. 2017; Hubbard et al. 2018; Newman et al. 2022) and fungal communities (Newman et al. 2022) in the rhizospheres of wildtype and mutant plants with either disrupted or abnormal circadian clocks. This showed that mutations which alter the function of the plant host circadian clock influence the composition of communities that assemble in the rhizosphere over the lifetime of a plant. Mutations in core components of the central oscillator such as *LHY*, *TIMING OF CAB EXPRESSION 1* (*TOC1*) and *ZEITLUPE* (*ZTL*) all led to altered bacterial and fungal community structure (Hubbard et al. 2018; Newman et al. 2022). This may impact plant health, as wild‐type Arabidopsis plants germinated earlier and were larger when inoculated with a soil slurry from wild‐type plants compared with *TOC1* or *ZTL* mutant plants (Hubbard, McMinn, and Weinig 2021). Furthermore, loss of function or overexpression of the *LHY* gene was shown to impact the relative abundance of endophytic, saprophytic, arbuscular mycorrhizal and pathogenic fungi (Newman et al. 2022). This indicates a role for the plant circadian clock in directing the composition of key functional groups within the microbiome over an extended growth period. This could occur as a result of changes to plant host immunity and through patterns of rhizodeposition. Changes in the balance between these functional groups resulting from aberrant clock function may cause increased incidence of plant disease or may impair interactions with beneficial microorganisms.

A key function of rhizosphere biota is their contribution to biogeochemical cycling and the provision of nutrients to plants. Plant growth is typically limited by availability of soil nitrogen (N) and phosphorus (P), and microbes have the potential to provide plants with access to N and P which reside in otherwise unavailable mineral and organic pools (Morgan et al. 2005). The extent to which rhizosphere biogeochemical cycling processes exhibit diurnal rhythmicity has received limited attention, but there is evidence for diurnal changes in the abundance of microbial transcripts involved in ammonium oxidation (amoA) (Nikolausz et al. 2008). Furthermore, N_2_O (Wu et al. 2021) emissions from diverse ecosystems are well known to show diurnal rhythmicity. A meta‐analysis showed that daytime peaks in N_2_O emissions are not coupled to temperature. Instead, they may be the result of enhanced denitrification and nitrifier denitrification during the daytime as a result of O_2_ depletion caused by the metabolism of rhizodeposits by heterotrophic microbiota (Wu et al. 2021). These findings suggest that microbial N‐cycling processes vary with the time of the day. This is important because the expression of plant genes involved in root NO_3_^–^ and NH_4_^+^ uptake, transport and assimilation is under circadian control. For example, transcript levels for the high‐affinity nitrate transporter (*NRT2*) increase in the day in Tobacco roots and decrease at night, and nitrate uptake is about 40% higher during the day than at night (Matt et al. 2004). The transcript for the *Arabidopsis* nitrate transporter *NRT1.7*, which is responsible for source‐to‐sink remobilisation of nitrate, was found to be peak at dusk, and this was followed by expression of the nitrate reductase gene *NIA2* during the night (Fan et al. 2009). The timing of microbial N‐cycling processes relative to plant metabolic processes may be important to maximise N‐availability at times of the day when plants are able to utilise this key nutrient. Disrupted temporal coordination between plant and microbial processes may result in impaired nutrition and may contribute to the reduced productivity of plants with abnormal circadian clocks (Green et al. 2002; Dodd et al. 2005).

Diurnal rhythmicity of CH_4_ emissions from soil has also been reported (Dai et al. 2019). As with N_2_O, this could be the result of diurnal changes in rhizodeposition and O_2_ availability (Dai et al. 2019), which results in anaerobic respiration and methanogenesis. It will be important to elucidate how plant rhizosphere processes influence diurnal rhythmicity in greenhouse gas emissions from soils because this has significant implications for understanding and predicting ecosystem‐climate feedbacks.

## 7 Perspectives and Future Research Directions

We propose that rhizosphere diurnal rhythmicity has evolved as a mutual adaptation between plants and rhizosphere microbes. It may promote fitness through various mechanisms including supporting plant and microbial adaptation to daily cycles of light and temperature, enhancing plant‐microbe nutrient exchange and improving plant defence against pathogens, driving co‐evolution of complex temporal dynamics in plant‐microbiome interactions. Understanding these processes holds promise for understanding ecosystem processes and for devising strategies to improve the sustainability of agricultural systems.

### 7.1 Spatial and Temporal Interactions and Drivers

The rhizosphere represents a rhythmically dynamic environment, and this is likely to be key to understanding its function and contribution to plant health. In this way, the rhizosphere microbiome shares similarities to the microbiome of the human gastrointestinal (GI) tract, which shows diurnal changes in composition and metabolism which are mediated by the host's circadian rhythm and feeding behaviour (Gutierrez Lopez et al. 2021). There are several factors which may direct the diurnal rhythmicity of the rhizosphere microbiome, and the interplay between them and the ways in which they are linked to both plant host and microbial circadian rhythms are still unclear. However, the key factor which controls diurnal changes in microbiome composition is likely to be the flux of exudate from plant roots into the rhizosphere during light periods, which drives the growth of heterotrophic microbes.

**Figure 3:**
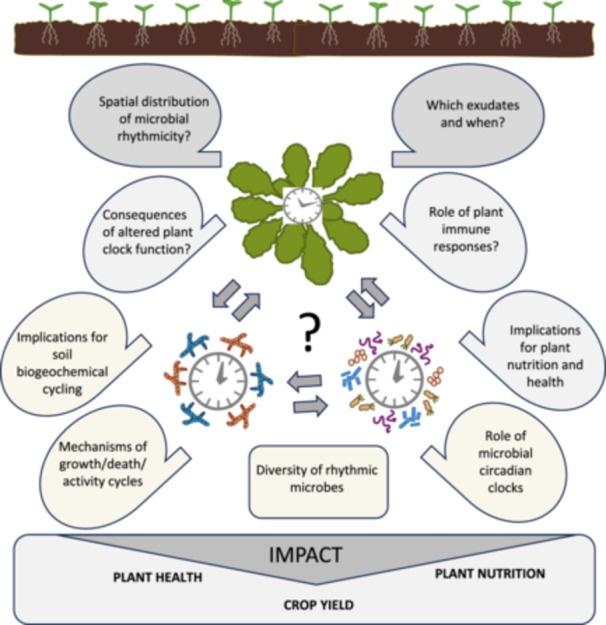
Key Knowledge gaps. Plant‐related questions are shown in grey bubbles and microbe‐related questions in yellow bubbles. [Color figure can be viewed at wileyonlinelibrary.com]

The significance of diurnal rhythmicity in rhizosphere microbiome composition for the host remains unclear. In humans, rhythmicity of GI microbiota results in rhythmic exposure of the intestinal epithelium to bacteria and their metabolites and these interactions show strong spatial variations across time (Thaiss et al. 2016). These spatial and temporal microbial rhythms feedback to affect the host's transcriptional, epigenetic and metabolic rhythms, which have the potential to influence the host's physiology and susceptibility to disease. Clearly there is a need to understand the cross‐talk and feedbacks which connect the rhizosphere microbiome and its plant host across diurnal timeframes and to understand their significance for plant health and nutrition, and soil biogeochemical cycling processes (Figure 3). Since evidence strongly links the plant circadian clock to these interactions there is a need to elucidate the pathways through which the plant clock operates, and the significance and implications of altered clock function for rhizosphere processes. This is particularly urgent since cultivation has resulted in altered clock function in many crop species (Bendix, Marshall, and Harmon 2015; McClung 2021).

The spatial organisation of rhizosphere microbial communities is poorly understood. Exudation occurs directly behind the root tip in the root expansion zone (Oburger and Jones 2018), and thus microbial growth cycles and microbe–host interactions are expected to be most intense in this region. The regulation of rhizosphere microbial rhythms may include conserved mechanisms linked to core circadian clock components which are shared across plant species and common responses to environmental cues. However, species‐specific differences in root exudation patterns suggest that some regulatory mechanisms are likely to be host‐dependent. Recently Loo et al. (2024) showed that root SWEET sugar transporters are spatially correlated with rhizosphere bacteria and that bacteria may induce accumulation of these transporters. Interestingly plant pathogens secrete virulence proteins which activate expression of specific SWEET transporters to support their own growth (Ji et al. 2022). While expression of leaf SWEET transporters may be controlled by the circadian clock (Hua et al. 2022), the rhythmicity of root SWEET transporters is currently unknown.

In common with most research on rhizosphere communities, studies of diurnal rhythmicity in the rhizosphere microbiome have pooled whole root systems, and this has the potential to dilute the magnitude and visibility of interactions which have discrete spatial zones. Accurate analysis of plant exudation is difficult, and many approaches used in the literature, including collection from plants grown in or removed to hydroponic systems, and extraction directly from soil containing plant roots are problematic and may not generate realistic data (Oburger and Jones 2018). Mass spectrometry imaging of tissues to characterise the 3D distribution of metabolites *in situ* could provide new opportunities to characterise exudate chemistry and also to obtain spatial data on the release and longevity of exudate components in the rhizosphere (Lohse et al. 2021).

Furthermore, research to date has focused on rhizosphere soil adjacent to the plant root. However, plant roots themselves support complex microbial communities, both on the surface of the root and as endophytes within the root itself. These populations are comprised of diverse taxa and include microbes which may exert positive or negative impacts on plant growth and development (Hilton et al. 2021; Lazar, Mushinski, and Bending 2022). These communities are likely to have far more intimate associations with the plant host than rhizosphere soil biota, and their growth may be strongly regulated by rhythmic changes in root metabolism, controlled by the host plant's circadian clock. Clearly, there is a need for research on model systems which retain the microbial complexity of natural rhizospheres while allowing sampling of defined root zones and tissues. For example, *Medicago truncatula* has been used extensively for symbiosis research with nitrogen‐fixing rhizobia (Gavrin and Scornack 2019). This interaction was recently found to be disrupted in mutants with altered circadian clock function (Kong et al. 2020; Achom et al. 2022), and this may represent an interesting system to explore how circadian rhythmicity of the plant host impacts on the success of interactions with bacterial endophytes.

Rhythmic production of defence compounds by the plant including salicylic and jasmonic acids could drive rhythmic inhibition and death of microbes, and additionally, antimicrobial peptides (AMP) such as thionins have received limited attention. In animals, the expression of AMP shows distinct diurnal rhythms, likely regulated by the circadian clock, which affects patterns of bacterial survival on the skin (Bilska et al. 2021). Similarly, in the model legume *Medicago truncatula*, the expression of many members of the nodule cysteine‐rich (NCR) family of peptides exhibits circadian rhythmicity (Achom et al. 2021). These peptides are known to play a role in controlling infection by nitrogen‐fixing endosymbionts (Farkas et al. 2014; Mikuláss et al. 2016), and the altered expression patterns of NCR peptides in LHY mutant plants may explain their reduced nodulation on infection by *Sinorhizobium melilotti* (Achom et al. 2021).

### 7.2 Characterising Microbial Rhythmicity and Interactions

Soil and rhizosphere microbial communities contain great diversity, which supports complex microbe–microbe interactions and feedbacks which ultimately control system properties such as biogeochemical cycling and plant growth. To date, most studies of diurnal rhythms in rhizosphere biota have focused on bacterial communities. Newman et al. (2022) demonstrated that fungal OTUs also show diurnal changes in composition and that rhythmic fungal OTUs may represent a greater proportion of the total community than is the case for bacteria. Since fungal communities are key components of the rhizosphere with well‐recognized effects on plant health (Gosling, Jones, and Bending 2016; Hilton et al. 2021) there is a need to ensure fungal communities are integrated into studies on rhizosphere microbial rhythmicity. In common with human microbiomes, there is a need to consider whether other microbial groups which inhabit the rhizosphere including viruses, archaea, protists and nematodes show rhythmic oscillations, and the interactions and feedbacks for rhizosphere functions and plant health (Frazier and Leone 2022).

The limited evidence available to date indicates that microbial diurnal rhythms are associated with a marked change in microbial abundance (Zhao et al. 2021, Hubbard et al. 2023), and furthermore, some populations show high relative abundance in the day and low relative abundance at night, while other populations display the reverse trend (Newman et al. 2022). Quantitative analysis of microbial abundance at the community level, and for specific rhythmic taxa, is an important knowledge gap. A range of markers including phospholipids and nucleic acids are available for the quantification of microbial biomass and growth rates, and their combination with stable isotopes such as ^2^H could be insightful (Caro et al. 2023). While the light‐dependent availability of exudates explains processes which drive growth and activity during the day, those processes which control death, growth and activity during the night are less clear. A range of factors could underlie these patterns. A key driver of microbial rhythmicity is likely to be shifts in the relative importance of readily assimilated exudates and biochemically complex soil organic matter and microbial necromass for supporting heterotrophic metabolism across diurnal timeframes. Changes in populations could thus result from starvation and death when exudate abundance is low, microbe‐microbe competition, as well as oscillation of cells between dormant and active states.

In marine systems, the activities of both protists and bacteriophages have marked diurnal cycles. Heterotrophic protists can display diel feeding cycles with increasing consumption of bacterial prey in the day relative to the night (Jakobsen and Strom 2004; Ng and Liu 2016; Arias, Saiz, and Calbet 2020; Deng, Cheung, and Liu 2020). Many of these rhythms persisted upon transfer to constant environmental conditions, indicating control by endogenous circadian clocks (Jakobsen and Strom 2004; Arias, Saiz, and Calbet 2020). While bacteriophages are unlikely to possess circadian clocks of their own, their populations show strong diurnal rhythmicity in marine systems, coupled with light‐driven cyclic growth of both photoautotrophic and heterotrophic bacterial hosts (Aylward et al. 2017). This is important because bacteriophage infection in the ocean turns over 20% of cyanobacterial biomass daily, with nutrients subsequently being released from necromass following degradation by heterotrophic bacteria. There is the intriguing possibility that these interactions could also occur in the rhizosphere, driving a diurnal rhizosphere microbial loop which releases nutrients from heterotrophic populations to support plant growth, as occurs in the oceans.

### 7.3 Unravelling the Broader Consequences and Significance of Rhythmicity

Hubbard et al. (2018) showed that the rhizosphere community associated with *toc1* clock mutant plants was distinct from that of wild‐type Arabidopsis, with evidence that this change in the rhizosphere microbial community was deleterious to plant health because wild‐type plants exhibited lower germination and reduced growth when sown onto soil previously planted with *toc1* mutants. Additionally, Newman et al. (2022) showed that a functioning circadian clock impacts the structure and function of the rhizosphere microbiome including taxa such as pathogens and endophytes which may directly influence plant health. There is a need for quantitative, mechanistic data on the way in which microbiome rhythmicity impacts plant health. Furthermore, diurnal rhythmicity in rhizosphere community composition and abundance could have broader effects on soil biogeochemical cycling processes. Shifts in microbial growth and activity patterns associated with altered C availability over diurnal timeframes have the potential to drive diurnal cycles of N and P assimilation into microbial biomass, resulting in competition with plant roots. While evidence suggests that transcription of plant nutrient transport proteins shows diurnal oscillation, there is a need to understand the extent to which this is associated with patterns of plant nutrient assimilation and its relationship with microbial nutrient mineralisation‐immobilisation processes associated with diurnal gradients in nutrient stoichiometry. This is a key knowledge gap given the strong evidence implicating rhizosphere processes in driving diurnal patterns of soil net N_2_O and CH_4_ emissions (Wu et al. 2021).

Some plant growth‐promoting microbes are known to improve the tolerance of plants to daily and seasonal biotic stressors such as pathogen and herbivore attacks, and to abiotic stressors such as drought and cold, through the production of phytohormones such as auxin (indole‐3‐acetic acid), cytokinin, abscisic acid and salicylic acid (Kudoyarova et al. 2019; Xu et al. 2023). On the other hand, the plant circadian clock gates (restrict) downstream signalling to prevent responses at the wrong time of the day (Robertson et al. 2008; Xu, Yuan, and Xie 2022). Bacterial production of phytohormones at the wrong time of the day would be a wasted resource for the plant host, and it would be interesting to determine whether bacteria that produce these hormones are more abundant at times of the day when the plant can respond to them. Microbial inoculants may therefore need to be tailored to the circadian clock of the specific crop variety for maximum effectiveness in agriculture. Belbin et al. (2019) showed that plants exhibit clock‐mediated circadian rhythmicity in their susceptibility to a herbicide and proposed the concept of agricultural chronotherapy, analogous to medical chronotherapy in which delivery of medicines is synchronous with circadian rhythms to maximise efficacy (Kaur et al. 2013). Diurnal variation in the composition of the rhizosphere microbiome could determine the success of soil‐based agricultural interventions, such as the establishment of plant growth‐promoting microbial inoculants, depending on the time of application.

## Data Availability

Data sharing is not applicable to this article as no new data were created or analysed in this study.
